# Gut Microbes as the Major Drivers of Rheumatoid Arthritis: Our Microbes Are Our Fortune!

**DOI:** 10.3390/microorganisms13020255

**Published:** 2025-01-24

**Authors:** Veena Taneja

**Affiliations:** Department of Immunology, Mayo Clinic, 200 First St. SW, Rochester, MN 55905, USA; taneja.veena@mayo.edu

**Keywords:** genetics, environment, transgenic mice, human leukocyte antigens, rheumatoid arthritis, host–microbiome interactions

## Abstract

Rheumatoid arthritis (RA) is an autoimmune disease with an unknown etiology. While certain genes provide strong susceptibility factors, the role of environmental factors is becoming increasingly recognized. Among genetic factors, human leukocyte antigen (HLA) genes, encoded within the major histocompatibility complex (MHC), have been linked to predisposition to RA, while among environmental factors, smoking, infections and diet are the major contributors. Genetic and environmental factors impact microbial composition in the host. Based on the dysbiosis observed in the gut and lung microbiome, a mucosal origin of RA has been suggested. However, proving whether genes or microbes provide a stronger risk factor has been difficult. Studies from RA patients and various mouse models, specifically humanized mice expressing HLA class II genes, have been instrumental in defining the role of environmental factors such as smoking and endogenous small intestinal microbes in modulating arthritis severity. The consensus based on most studies support an interaction between host genetic and environmental factors in the onset and severity of disease. However, until now, no microbial markers for disease prognosis or treatment efficacy have been available. Here, the role of gut microbes as markers of disease severity, and the potential for using endogenous commensals for modulating immune responses to suppress inflammation in the context of genetic factors, are discussed.

## 1. Introduction

Uncovering the role the microbiome plays in human health, along with technical advances in sequencing the microbiota, has led to an understanding that we live in symbiosis with our microbiome [1]. However, that symbiosis is dependent on many external factors including diet, environment, lifestyle, personal hygiene, and infections, among others [2,3,4]. Besides external factors, host genetics also might play a role in determining the microbial composition of an individual [5,6,7,8]. ‘You are what you eat’ is a common expression used to describe the healthy and non-healthy impact of our diets. While our environment influences many physiological functions directly or indirectly, the question of why only some individuals in the same environment are adversely afflicted by several of these factors is unclear. Also, individuals can suffer from dissimilar effects of the same environment. For example, smoking does not cause pathology for everyone exposed to it. The major reasons for these differences have been attributed to genetics and epigenetics, which can turn genes on or off. Indeed, epigenetic differences in sex chromosomes account for many autoimmune diseases and cancers, with autoimmunity observed more often in women and cancers in men [9]. Humans harbor many polymorphic genes that are involved in producing enzymes and molecules which impact epigenetics and various physiological functions. Post-translational modifications such as DNA methylation and histone modifications are common processes required to clear infections. Some of the enzymes required for these modifications are produced in the gut with the help of the microbes residing in the gut, which help in the biotransformation of various products for their use by the host. In addition, microbes can cause alterations in the gene expression of the host via epigenetics, thus connecting the environment with the immune system [10,11,12].

The human body is host to trillions of microbes on various mucosal surfaces. The intestine provides the biggest mucosal surface and carries the maximum number of microorganisms, including microbes as well as fungi, viruses and archaea, which support physiological functions locally and systemically. Colonization of the intestine begins in utero and continues throughout early childhood until the age of 3 years, after which it stabilizes based on exposure to external factors [13,14]. The gut microbial composition evolves in microbial diversity and richness in symbiosis with the host, dictated by an individual’s environment, and bacteria acquired during birth and in utero are no longer represented predominantly during adulthood, though some maternal microbial clades can be present, suggesting a core microbiome [14,15]. The microbiome of an individual is like a unique fingerprint which has enormous impact on various immune functions locally as well as systemically.

What is a healthy microbiome? Further, does environment define the microbial composition of an individual, or do genetic factors determine microbial composition and enrichment in the gut? The published literature does not provide a clear picture, as major work has been conducted in animal models. For humans, the most easily available sample for determining intestinal microbiome is stool. Sequencing for bacteria for defining microbial diversity and composition in the upper gut requires biopsy, which is not feasible in most conditions except for certain gastrointestinal diseases. However, stool microbiota provides an incomplete picture of the microbial diversity, function and role in various conditions, as microbes in the upper gut interact with the immune system and could play a crucial role in intestinal and extraintestinal diseases. Mouse models of diseases have highlighted the importance of small intestinal microbes in various conditions including arthritis, as discussed below [16,17,18]. Studies with germ-free mice are good models with which to study specific mechanisms but are not ideal as humans and animals do not live in germ-free conditions. Even though advances in technology have helped us understand the crosstalk between the gut microbiome and immune system, there is still limited information on its use as a marker of disease and treatment efficacy. This brief review focuses on the role of intestinal microbes in inflammatory diseases such as RA and the use of intestinal commensals for suppressing inflammation.

## 2. Interaction Between Environmental Factors and Genes in Autoimmunity

Dominant mutations in genes contribute to the overexpression or deletion of proteins, which can result in associated conditions; for example, mutations in protein tyrosine phosphatase non-receptor type 22 (PTPN22) have been linked to autoimmunity [19,20]. Although the direct impact of genetic abnormalities associated with diseases is understandable, epigenetic changes that cause such conditions are difficult to prove. Recent technologies like whole-genome sequencing is providing a leap forward for such conditions. Various gene therapies are being developed for inherited conditions, where genes for the correct proteins/molecules are delivered using vectors, stem cells or liposomes to direct them to make proteins/molecules that can correct the body’s response [21,22].

The question is whether all genes are amenable to alterations for generating correct responses? Genes within the major histocompatibility complex (MHC) encoding for human leukocyte antigens (HLA) are the most polymorphic region in the human genome. HLA molecules present extracellular pathogen-derived peptides to generate an immune response to clear infections. Recent studies on the severity of viral infections such as SARS-Cov-2 and its resistance and clearance have shown a correlation with HLA polymorphism [23,24], and, though not yet confirmed, long-term side effects may also be linked to genetic factors including the HLA genes. One of the reasons for this could include a bystander effect of a robust response or a molecular mimicry of pathogenic epitopes with a self-epitope ensuing an autoreactive response when presented with HLA molecules. Indeed, patients with various autoimmune diseases harbor certain HLA genes more often than healthy populations [25,26]. However, not all individuals with disease-susceptible HLA genes develop diseases. HLA-DR occurs in linkage with HLA-DQ genes, and while certain combinations provide a higher risk of developing pathologic conditions, they may be crucial for pathogen clearance. One such HLA gene is DQ8, which is common in various populations but has been linked to many inflammatory diseases, including rheumatoid arthritis, celiac disease, multiple sclerosis, diabetes, etc. [25]. HLA-DQ8 provides a unique perspective on the role of genetic factors in health and disease. It occurs in linkage with DR4 genes and, based on the DR4 subtype, it forms a susceptible haplotype present in many conditions including RA. DQ8 is a promiscuous molecule and evolutionarily might have an advantage of presenting various pathogenic epitopes, as well as being able to present post-translationally modified epitopes [27]. However, altering such genes is not a feasible approach for treating inflammatory conditions. For most conditions, only the downstream events of inflammatory immune responses are blocked by treatments using biologics, NSAIDs and immunosuppressives.

## 3. Rheumatoid Arthritis—A Consequence of Interactions Between Environment and Genetic Factors

Rheumatoid arthritis is a multifactorial autoimmune disease with an unknown etiology which can cause joint damage and disability [28,29]. A familial aggregation of the disease underscores the role of genetic factors [30,31]. The genetic underpinnings of RA have been explained by an increased occurrence of certain HLA class II genes and other non-MHC genes in patients [32,33,34]. Among HLA class II genes, HLA-DRB1*0401 has been linked to RA in multiple studies. However, ethnic variability in the HLA genes has led to variable associations with RA based on the ethnic population. This led to the proposition of the “shared hypothesis” (SE), according to which HLA alleles sharing the third hypervariable region with the DRB1*0401 were associated with RA in most populations. Patients carrying ‘SE’ demonstrated stronger links with seropositive RA [35]. This hypothesis assumed that HLA molecules in SE present an arthritogenic antigen. However, due to a lack of a specific arthritis-causing antigen, it has been difficult to prove the causative impact of the associated HLA-alleles. Since HLA-DR and DQ occur in linkage, later studies determined the role of HLA-DQ3 (DQ7 and DQ8), known to be in linkage with DR4. These studies linked severe disease with the presence of DQ7 and DQ8 in two different populations [36,37]. The observations were tested using humanized mice expressing HLA-DQ8 and the haplotype DRB1*0401/DQ8, which implicated the role of HLA-DQ in arthritis. Using mice carrying the RA-resistant allele HLA-DRB1*0402 and the haplotype *0402/DQ8 in an arthritis model demonstrated that the DQ loci could be prominent in disease predisposition, while DR4 subtypes acted as modulators of inflammatory response and sex-specificity [9,38,39,40].

While initial studies used serological means to define the link between HLA-DRB1/DQB1 and RA, later studies mapped single nucleotide polymorphisms (SNPs) [41,42]. Using large cohorts and SNPs, the association between the HLA region and RA were observed to be independent of the shared epitope [43]. Genome-wide association studies (GWASs) have associated 106 genes with susceptibility to RA [44] and a difference in genetic associations between seropositive and seronegative patients, suggesting it is a polygenic condition. The fact that genetic variability can differentiate seropositive and seronegative disease suggests that it can be used to characterize disease heterogeneity. The fine mapping of the GWAS identified variants of some causal loci, such as PTPN22 and Tyk2 [45]. Further studies have defined epigenetic marks in specific cell types to distinguish causal versus non-causal variants associations with RA [46]. With the advent of profiling gene expression and the functional aspects of a single cell, it is possible to define the causal cell-specific phenotypes in the synovium and periphery. Since many environmental factors can cause epigenetic changes, an implicated interaction between genetic and environmental factors is integral to the onset and progression of the disease. Among the environmental factors, smoking has been studied extensively and has been linked to seropositive RA [47]. The consequence of smoking is the increased presence of citrullinated peptides that can be presented by HLA-DRB1*04 as well as HLA-DQ8 [48,49,50], generating a cellular and humoral immune response to self-antigens. Smoking has been linked to higher levels of anti-citrullinated antibodies and with severe seropositive disease, specially in DRB1*0401-positive patients and SE positive individuals. However, not all smokers develop RA, and healthy individuals can also be positive for ACPA [47,51].

The interaction between the genetically susceptible HLA molecules and smoking in exacerbation of RA was elucidated in mouse models where humanized mice expressing HLA-DQ8 and DRB1*0401 were exposed to cigarette smoke and induced collagen-induced arthritis (CIA). HLA-DQ8 mice exposed to cigarette smoke developed severe CIA with antibodies to citrullinated proteins and rheumatoid factor (RF) along with lung pathology [48,52]. On the other hand, DRB1*0401 mice did not develop severe disease, but showed sex-specific bias in arthritis [38,39,40]. The exposure of *0401 mice, as well as mice carrying the RA-resistant gene, DRB1*0402, to cigarette smoke generated a similar response to native and citrullinated self-peptides [50], even though *0402 mice did not develop arthritis, suggesting an interaction between the impact of HLA genes and environmental factors on RA onset [53].

Besides smoking, an infectious etiology has also been proposed for RA onset. An increased presence of certain pathogens, such as *Proteus mirabilis* and *Porphyromonas gingivalis*, as well as antibodies to certain viruses such as parvovirus and Epstein–Barr virus (EBV), are present in patients with RA [54,55,56,57], linking infections to RA onset. Some oral microbes such as *P. gingivalis* and *P. butyrica* carry the enzyme peptidyl arginine deiminase (PAD) required for citrullination and can contribute via the citrullination of self-proteins [58,59,60]. One can speculate that in individuals with genetic susceptibility, exposure to infections might help break tolerance to self-proteins in case of molecular mimicry with pathogen-derived products. However, the limitation of this hypothesis has been a lack of a specific pathogen in various ethnic groups that could explain the onset of RA. Importantly, environmental factors such as smoking alter the lung microbiome as well as the intestinal microbial composition [61]. RA patients display dysbiosis in the lung lacking *Prevotella* and *Porphyromonas*. [62]. In addition to intestinal and lung dysbiosis, alterations to the oral microbiome [60,63] and subgingival microbiome, and the presence of oral microbes such as *P. gingivalis* in the synovial fluid of RA patients [64], suggest that RA-linked microbes can cause leaky gut and travel to the joints.

These observations led to the concept of a mucosal origin of RA. However, the contribution of various mucosal sites is still unknown. Indeed, mucosal sites share microbes and, dependent on the origin of the break of tolerance, resident microbes of specific mucosal sites may be involved. Since the intestinal microbiota is the most abundant and diverse and is involved in controlling immune response locally and systemically, it has garnered the most attention for a link between the microbiome and diseases.

## 4. Microbiome in RA

Humans are host to trillions of microbes that form an ecosystem which is required for the homeostasis of the immune system. Disruption to that ecosystem can cause dysbiosis with abnormal immune system function. Since the gut contains the maximum number of microorganisms, alteration in microbial composition due to environmental factors can disrupt the homeostatic milieu in the gut. It can also provide an opportunity for the expansion of a pathogenic microbe. Subsequently, this could lead to becoming involved in continuous low-grade inflammation, which can easily break tolerance in the event of insult to the immune system. Indeed, preclinical autoreactivity can be present in patients with RA for up to 10 years prior to transitioning from asymptomatic to clinical onset of disease. Smoking has been suggested as one of the factors that can cause preclinical autoreactivity; one can speculate that endogenous factors such as the gut microbiota might play a role in preclinical autoreactivity.

While diet, lifestyle age and hygiene show a strong impact on intestinal microbial composition, immunogenetic control of the microbiome has been established [5], suggesting that genetic factors and immunity might determine the genus and strains that can survive and live in harmony with an individual’s immune system. Thus, an individual carrying a genetically susceptible RA-associated HLA allele might harbor opportunist pathogens which can expand under certain circumstances. This concept was confirmed in RA patients where established seropositive RA patients with severe disease showed an expansion of *Eggerthella lenta* and an altered metabolic profile [63,65]. Whether *E. lenta* is causative or contributes to RA pathogenesis is unknown. A mouse model was utilized to investigate whether *E. lenta* is causative for arthritis. Humanized mouse models confirmed the contribution of *E. lenta* in preclinical autoreactivity as naïve DQ8 mice colonized with *E. lenta* produced rheumatoid factor (RF) and generated responses to peptides derived from type II collagen (CII). DQ8 mice with CIA gavaged with or without *E. lenta* generated responses to *E. lenta*, suggesting a cross-reactive response to endogenous protein as a causative factor. A comparative analysis showed that certain *E. lenta* proteins share homology with sequences in CII [66]. Further, arthritic mice gavaged with *E. lenta* developed CIA with an earlier onset and higher antibody levels similar to observations in RA patients. In addition, *E. lenta* expansion caused dysbiosis with reduced diversity and metabolic alterations which resembled that of aged individuals. However, *E. lenta* in naïve mice, without an activated immune response, did not cause arthritis, suggesting a second insult to the immune system can result in pathogenesis in genetically susceptible individuals with preclinical autoreactivity.

It is difficult to test the metabolic profile of RA patients before they become symptomatic, and since autoreactivity can occur much before actual transition to joint involvement, mouse models are necessary to determine when and which metabolic changes are important for pathogenicity. For this, DQ8 mice were tested for alterations in the metabolic profile specific to *E. lenta* expansion by comparing the untargeted metabolic profile before and after *E. lenta* gavage in arthritic mice. *E. lenta* expansion caused a significant increase in secondary bile acids and their receptor, sphingosine 1 phosphate receptor 1 (S1PR1), which has been linked to gut inflammation and the regulation of lipid metabolism, as well as the senescence of fibroblasts [67,68,69,70]. On the other hand, there was a significant decrease in amino acids, including citrulline and tryptophan, required for protein synthesis, while tryptophan-derived indoles were increased [66]. Reduced tryptophan led to a reduced de novo production of Nicotinamide Adenine Dinucleotide (NAD), known for its anti-inflammatory effects and promotion of arginine biosynthesis, thereby reducing Th17 differentiation and inflammation [71,72,73,74]. Reduced NAD levels in *E. lenta* gavaged mice were associated with an increase in IL-17-producing T and B cells [66]. Indeed, microbiota-derived indoles have been linked to CIA in mice [75]. Besides augmenting disease severity by altering microbial composition and function, *E. lenta* also skewed the autoreactive response in a sex-specific manner, with more antibodies in female mice and seropositive female RA patients. These data clearly implicate that in endogenous opportunist bacteria, which are otherwise involved in metabolism, *E*. *lenta* metabolizes arginine, which can become pathogenic under some circumstances. Further, it is possible that gut commensals contribute to the sex-specificity of autoimmune diseases.

The other examples of the contribution of pathogenic endogenous bacteria include *Prevotella copri*, which was observed in new-onset RA patients (NORA) [76], and *Subdoligranulum didolesgii* isolated from at-risk individuals, which was shown to trigger joint swelling, as well as autoantibodies to CII in germ-free mice [77]. The dysbiosis and expansion of pathogenic microbes can increase intestinal permeability, leading to an egress of bacterial products. RA patients harbor antibodies to peptides derived from *P. copri* [78]. However, the enrichment of genes in *P. copri* and its function was dependent on the diet, suggesting an impact of diet on microbial composition and functional status [79], which can contribute to the local milieu.

**Figure 1:**
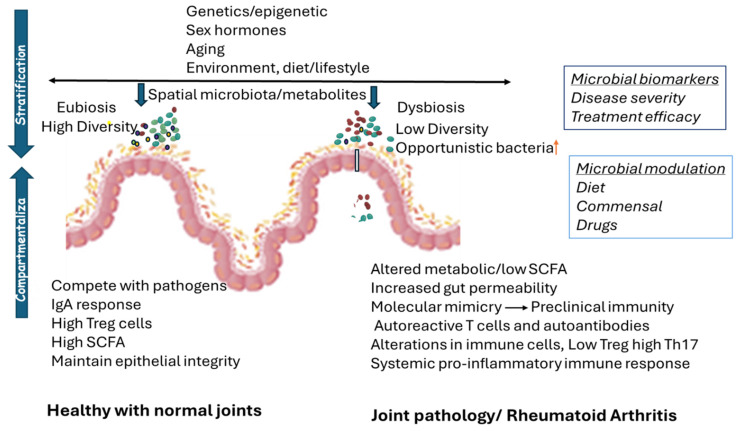
Mucosal origin of preclinical autoreactivity and the role of microbes in the pathogenesis of rheumatoid arthritis (RA). Intestinal microbial diversity is shaped by genetic factors encoded within the major histocompatibility complex (MHC-II) and environmental factors including lifestyle (smoking, diet, exercising), infections and geographic location, among others. Besides these factors, epigenetics, sex hormones, and an aging immune system also contribute to microbial composition, thereby establishing a unique microbiome for each individual. Intestinal microbial composition is based on the requirement and function of the region generating spatial microbial diversity. Individuals with RA-susceptible genes may harbor opportunistic pathogens which, under certain circumstances, cause the dysbiosis and expansion of those endogenous pathogens accompanied with reduced diversity. This can alter the metabolic profile with an increase in certain metabolites and a decrease in short chain fatty acids (SCFA) required for a healthy gut. Microbial/metabolic dysbiosis impacts the immune cell profile, causing inflammatory milieu in the gut, thereby increasing gut permeability. If the endogenous pathogen has molecular memory with a self-peptide, it can break tolerance and generate preclinical autoreactivity. Activated immune cells and luminal products can extravasate and cause systemic inflammation. In the joints, these activated cells can trigger the local immune system, causing inflammation and epitope spreading, resulting in a transition from asymptomatic to symptomatic arthritis and pathology. Knowledge of the endogenous pathogen can help define biomarkers of disease onset and progression. In healthy individuals, symbiosis between the immune system and gut microbes producing SCFAs helps with epithelial cell repair and the generation of T regulatory cells, keeping pathogens and inflammation in check. Besides the gut microbiome, other mucosal surfaces such as the oral cavity and lungs may also be involved suggesting a mucosal origin of preclinical asymptomatic autoreactivity. Treatments and diets can alter the gut microbiome and partially restore the microbiome, thus suppressing inflammation. The use of endogenous commensals with probiotic-like properties, such as *Prevotella histicola*, can generate eubiosis and help maintain T regulatory cells in the gut, which can reverse the inflammatory response and help with reducing symptoms associated with arthritis. Endogenous commensals that imitate the effect of biologics can further aid in reducing the dose of immunosuppressive drugs used for treating patients, thus helping to reduce side effects.

There are many studies that have described dysbiosis in RA and the presence of specific taxa. How this expansion of a taxa contributes to the onset of RA is still unclear, though mouse models have provided information on their contribution to the dysregulation of immune system and preclinical autoreactivity (Figure 1). However, what causes the break in tolerance from preclinical reactivity resulting in symptomatic disease is still being investigated.

## 5. Gut Commensals as Predictors and Probiotics

Since intestinal microbes had been indicated in pathogenesis, the question arose whether commensals can also be used to predict treatment efficiency or disease progression. Measures of clinical disease activity are used regularly to assess patients’ disease status; however, there are no available biomarkers that predict disease progression or treatment efficacy. Recent studies have demonstrated an increase in microbes of Actinobacteria phylum, specially genus *Collinsella* and *Eggerthella* [63,65], which correlated with severe disease. In an effort to ascertain microbiota as predictors of treatment efficacy, RA patients treated with Methotrexate (MTX) were followed for alterations in microbial composition and response to treatment [80,81]. Observations demonstrated that MTX responders had diverse microbiota, which were partially normalized and differential compared to non-responders. Clinical response was associated with an increase in Prevotellaceae family presence in established RA patients and OTUs of *Prevotella* species in new-onset RA patients [80,81]. Clinical improvement was linked to sugar metabolism, fatty acid and beta-oxidation and biotin biosynthesis, all of which are functions that require intestinal microbes [80]. These studies point to microbial and metabolic markers as predictors of treatment; however, studies in bigger cohorts and ethnic groups need to be conducted.

The major function of gut commensals is to harvest energy from the diet and contribute to the immune system by producing short chain fatty acids (SCFA); it is possible to define the genus that can be used as a probiotic for treating RA. Faecalibacterium is a predominant butyrate, SCFA and producer in healthy humans which is reduced in RA patients. A low abundance of *F. prausnitzii* causes a decrease in butyrate production [65,82] and production of IL-10, an anti-inflammatory cytokine [83]. *F. prausnitzii* modulates the immune response by suppressing NFkB [83,84,85]. Patients with RA harbor higher levels of bacteria that consume butyrate, which has been linked to disease severity [86]. Systemic autoimmunity has been associated with low levels of butyrate-producing bacteria [87]. Butyrate is used by colonocytes for epithelial layer repair, thus it might contribute to improving intestinal epithelial permeability. Indeed, supplementation with butyrate has shown potential therapeutic effects on suppressing inflammation [87]. However, since Faecalibacterium is present in abundance in humans, its supplementation may have limited benefits.

Besides Faecalibacterium, other common genera like *lactobacilli* have been studied for treating RA. In a randomized controlled clinical trial (RCT), a mixture of *lactobacillus* species, *Lactobacillus rhamnosus* and *Lactobacillus reuteri*, or probiotic *Lactobacillus casei*, were used to treat RA patients, which led to a reduction in proinflammatory cytokines IL-1α, IL-6, IL-12 and TNF-α [88], as well as a decrease in swollen joints [89]. The administration of *Lactobacillus casei* in a mouse model of arthritis also demonstrated reduced antibodies, leading to a decrease in arthritis incidence [90]. There are many potential mechanisms by which probiotics can provide health benefits to the host [91]. Probiotics can compete with pathogens, produce SCFAs for epithelial cell repair to reduce gut permeability, reduce inflammatory cytokines, generate T regulatory cells, leading to the production of IL-10 and dampening inflammation, suppress proliferation and alter microbial composition, which supports good health. However, not all mechanisms have been explored, and, due to individual and spatial microbial variability, no probiotic has yet not shown benefits in all patients.

In humans, most studies are conducted with fecal samples as upper-gut bacteria are difficult to access and require biopsy. Microbes can have niche-specific impact, as microbial diversity and functions along the intestine are based on nutritional requirements. For example, *Prevotella* is a predominant genus in humans; however, based on the niche, variable properties have been observed. Fecal *P. copri* has been linked to NORA patients [76], while duodenal *Prevotella* has been associated with immunomodulatory and probiotic properties in arthritis [17,82,92]. A novel *Prevotella histicola* isolated from duodenal biopsy when administered to arthritis-induced DQ8 mice in prophylactic and therapeutic protocols showed significant reduction in arthritis incidence and disease severity [17,82,92]. The suppression of arthritis severity was accompanied by alterations in intestinal and fecal microbial composition, with an increase in butyrate producers and tight junction proteins, thus improving gut epithelial integrity [93]. Since upper-gut bacteria are more likely to interact with the immune system, the study also analyzed alterations in immune cells caused by *P. histicola* in arthritic DQ8 mice. An increase in T regulatory cells and myeloid suppressors leading to an increase in IL-10 production with reduced autoantibodies was observed in *P. histicola*-treated arthritic mice as compared to non-treated arthritic DQ8 mice. The novel *P. histicola* colonized the duodenum of mice and augmented the production of butyrate, and also has genes involved in biotin and folate synthesis and metabolizes sugars, factors observed to be associated with treatment efficiency [80,92]. Recent data have shown that duodenal *P. histicola* suppresses arthritis severity in DQ8 mice, mimicking the efficacy of the TNF inhibitor (TNFi) that is a commonly used biologic for treating RA [94]. Additionally, *P. histicola* could prevent the flare of disease activity caused by the discontinuation of TNFi.

These observations suggest that intestinal commensals and their function can be used as markers for predicting disease progression and for preventing disease severity. It might be possible to use commensals such as *P. histicola* to lower drug doses to prevent side effects. Since *P. histicola* is reduced in autoimmune diseases and is present in healthy humans, treatments might not cause serious side effects. Whether it is the genetic profile of an individual which leads to a deficiency of *P. histicola* in patients, or the impact of environmental factors is unknown.

In a vegetarian diet specifically, fiber is known to increase the abundance of the *Prevotella* genus [95]. Is it possible to increase potential beneficial commensals by changing diet to help suppress inflammation? Indeed, a high-fiber diet has been demonstrated to alleviate type 2 diabetes by altering the gut microbiome [96]. While the mediterranean diet, which is known to include fiber and healthy fatty acids, has shown beneficial effects in arthritis [97,98], there is no diet that has been shown to prevent RA, though it can reduce inflammation [99]. Since dietary requirements can differ based on sex and have sex-specific effects, this needs to be evaluated in patients.

## 6. Genes and Microbes Are Drivers of Health

What is the connection between inflammatory diseases, infectious diseases, autoimmunity and cancer, and is there a common strategy for their treatment? If we presume that our genes control the immune response, microbiome, metabolome, epigenome and proteome, we can attempt to modify the products, but not the genes, or at least not all genes. We can control gene products; for example, if our food habits are healthy, even though an individual harbors susceptibility genes, the microbiome can be altered, albeit transiently, modifying the resultant immune response and related functions. The senescence of the immune system is considered a hallmark of RA. The impact of microbial diversity based on age and its interaction with the immune system in health and disease have been reviewed, with most studies being in agreement regarding the impact of microbes on systemic immunity during aging and in extraintestinal diseases [3,59,100].

Microbes can have niche-specific effects, and based on the diet, their gene enrichment may define the impact on the immune system, as indicated by the *Prevotella* genus in RA, where one *Prevotella* species is associated with RA onset while another is linked to treatment efficacy, as well as suppressing inflammation [17,59,76,81]. Since *P. histicola* simulates the action of a biologic drug, the use of commensals as probiotics or prebiotics for reducing inflammation is a possibility, as shown by [18,94]. The role of the diet in healthy aging by modulating the diet and the microbiota has been explored in a comprehensive review [2]. An epigenome–microbiome axis will show that genetic factors and microbial diversity interact. An individual with disease-susceptible genes might harbor certain opportunist commensals which in healthy conditions behave normally, but under certain circumstances such as stress or infections, can expand, resulting in microbial/metabolic dysbiosis.

## 7. Conclusions and Future Perspectives

This review is focused on the role of the gut microbiome and genetic factors in inflammatory RA in patients and mouse models of arthritis. The studies presented here indicate the role of genetic factors, specifically HLA genes and altered microbial diversity in RA pathogenesis. Dysbiosis with the involvement of specific pathogens may be different in various populations, and studies have shown the role of endogenous microbes in preclinical autoreactivity, as well as markers of treatment efficacy [3,6,59,101]. Microbial modulation using endogenous commensals can suppress inflammation in a similar way to biologics. There is an exponential growth of studies describing microbial associations with RA in various populations; 255 original research and 204 reviews, as per a bibliometric analysis performed last year [101]. This review is focused on the impact of microbiota in RA and limits the discussion on the role of hormones, sex and other factors, including diet, race and senescence, that are known to impact RA pathogenesis.

Humans are like a colorful crayon box, with different colors and unique genetic makeup. Women are having children at a later age, does this trend make them more vulnerable to immune disorders? Social evolution may be faster to recognize than genetic evolution. Climate change, zoonotic transmission of diseases, advances in technology and artificial intelligence could be related to social evolution. Over a period of time, it might be possible to find subtle genetic changes that can explain social evolution. Generally, investigative research is very focused, thus interactions among various genes and their functions in various organs are bound to be missed. Though genome sequencing can provide genetic associations with diseases, the question is about how we find safer treatments with lower side effects for all conditions. One method could be to achieve eubiosis via modulation of the gut microbiota. This can be accomplished by various means, including the use of selective probiotics, prebiotic supplementation, dietary changes and fecal transplants [82]. One such commensal is *P. histicola*, which reduces inflammation in a way similar to TNFi, without causing any pathology [17,92,94]. Since the commensal is a resident of various mucosal surfaces in healthy humans, it is less likely to cause significant side effects. However, for using endogenous commensals as probiotics, viability and delivery need to be optimized. The use of genetically modified commensals expressing anti-inflammatory molecules can be another strategy. Another option could be the use of metabolites, microbe-derived SCFAs and the byproducts generated by the metabolization of dietary factors, as well as those generated by modifications of host proteins such as secondary bile acids. However, one therapy may not work for all patients due to the multifactorial nature of disease. The answer may lie in combining the power of new technologies such as genome sequencing with spatial transcriptomics, epigenomics, metabolomics and metagenomics to identify specific markers and develop strategies to target them in various conditions. These technologies can be revolutionary in providing specific cells and pathways for developing treatment strategies to suppress inflammation. Considering the heterogeneity of diseases and the variability of genetic and geographic factors in various ethnic populations, future studies will need to be conducted in large cohorts from all over the world. Adapting microbial and microbial-derived factors as markers of disease and for treatment efficacy in the clinic will require a uniform strategy.

## References

[1] Chow J., Lee S.M., Shen Y., Khosravi A., Mazmanian S.K. (2010). Host-bacterial symbiosis in health and disease. Adv. Immunol..

[2] Golshany H., Helmy S.A., Morsy N.F.S., Kamal A., Yu Q., Fan L.P. (2024). The gut microbiome across the lifespan: How diet modulates our microbial ecosystem from infancy to the elderly. Int. J. Food Sci. Nutr..

[3] Donald K., Finlay B.B. (2023). Early-life interactions between the microbiota and immune system: Impact on immune system development and atopic disease. Nat. Rev. Immunol..

[4] Lopez-Otin C., Blasco M.A., Partridge L., Serrano M., Kroemer G. (2023). Hallmarks of aging: An expanding universe. Cell.

[5] Marietta E., Rishi A., Taneja V. (2015). Immunogenetic control of the intestinal microbiota. Immunology.

[6] Hall A.B., Tolonen A.C., Xavier R.J. (2017). Human genetic variation and the gut microbiome in disease. Nat. Rev. Genet..

[7] Qin Y., Havulinna A.S., Liu Y., Jousilahti P., Ritchie S.C., Tokolyi A., Sanders J.G., Valsta L., Brozynska M., Zhu Q. (2022). Combined effects of host genetics and diet on human gut microbiota and incident disease in a single population cohort. Nat. Genet..

[8] Zhernakova D.V., Wang D., Liu L., Andreu-Sanchez S., Zhang Y., Ruiz-Moreno A.J., Peng H., Plomp N., Del Castillo-Izquierdo A., Gacesa R. (2024). Host genetic regulation of human gut microbial structural variation. Nature.

[9] Taneja V. (2018). Sex Hormones Determine Immune Response. Front. Immunol..

[10] Zhao Y., Zhong X., Yan J., Sun C., Zhao X., Wang X. (2022). Potential roles of gut microbes in biotransformation of natural products: An overview. Front. Microbiol..

[11] Thaiss C.A., Levy M., Korem T., Dohnalova L., Shapiro H., Jaitin D.A., David E., Winter D.R., Gury-BenAri M., Tatirovsky E. (2016). Microbiota Diurnal Rhythmicity Programs Host Transcriptome Oscillations. Cell.

[12] Pepke M.L., Hansen S.B., Limborg M.T. (2024). Unraveling host regulation of gut microbiota through the epigenome-microbiome axis. Trends Microbiol..

[13] Koenig J.E., Spor A., Scalfone N., Fricker A.D., Stombaugh J., Knight R., Angenent L.T., Ley R.E. (2011). Succession of microbial consortia in the developing infant gut microbiome. Proc. Natl. Acad. Sci. USA.

[14] Martino C., Dilmore A.H., Burcham Z.M., Metcalf J.L., Jeste D., Knight R. (2022). Microbiota succession throughout life from the cradle to the grave. Nat. Rev. Microbiol..

[15] Valles-Colomer M., Blanco-Miguez A., Manghi P., Asnicar F., Dubois L., Golzato D., Armanini F., Cumbo F., Huang K.D., Manara S. (2023). The person-to-person transmission landscape of the gut and oral microbiomes. Nature.

[16] Gomez A., Luckey D., Taneja V. (2015). The gut microbiome in autoimmunity: Sex matters. Clin. Immunol..

[17] Marietta E.V., Murray J.A., Luckey D.H., Jeraldo P.R., Lamba A., Patel R., Luthra H.S., Mangalam A., Taneja V. (2016). Suppression of Inflammatory Arthritis by Human Gut-Derived Prevotella histicola in Humanized Mice. Arthritis Rheumatol..

[18] Bodkhe R., Balakrishnan B., Taneja V. (2019). The role of microbiome in rheumatoid arthritis treatment. Ther. Adv. Musculoskelet. Dis..

[19] Higuchi T., Oka S., Furukawa H., Tohma S. (2024). The contributions of deleterious rare alleles in NLRP12 and inflammasome-related genes to polymyalgia rheumatica. Sci. Rep..

[20] Bottini N., Musumeci L., Alonso A., Rahmouni S., Nika K., Rostamkhani M., MacMurray J., Meloni G.F., Lucarelli P., Pellecchia M. (2004). A functional variant of lymphoid tyrosine phosphatase is associated with type I diabetes. Nat. Genet..

[21] Verma I.M., Weitzman M.D. (2005). Gene therapy: Twenty-first century medicine. Annu. Rev. Biochem..

[22] Mashel T.V., Tarakanchikova Y.V., Muslimov A.R., Zyuzin M.V., Timin A.S., Lepik K.V., Fehse B. (2020). Overcoming the delivery problem for therapeutic genome editing: Current status and perspective of non-viral methods. Biomaterials.

[23] Augusto D.G., Murdolo L.D., Chatzileontiadou D.S.M., Sabatino J., Yusufali T., Peyser N.D., Butcher X., Kizer K., Guthrie K., Murray V.W. (2023). A common allele of is associated with asymptomatic SARS-CoV-2 infection. Nature.

[24] Tavasolian F., Rashidi M., Hatam G.R., Jeddi M., Hosseini A.Z., Mosawi S.H., Abdollahi E., Inman R.D. (2021). HLA, Immune Response, and Susceptibility to COVID-19. Front. Immunol..

[25] Mangalam A.K., Rajagopalan G., Taneja V., David C.S. (2008). HLA class II transgenic mice mimic human inflammatory diseases. Adv. Immunol..

[26] Mangalam A.K., Taneja V., David C.S. (2013). HLA class II molecules influence susceptibility versus protection in inflammatory diseases by determining the cytokine profile. J. Immunol..

[27] Chow I.T., Gates T.J., Papadopoulos G.K., Moustakas A.K., Kolawole E.M., Notturno R.J., McGinty J.W., Torres-Chinn N., James E.A., Greenbaum C. (2019). Discriminative T cell recognition of cross-reactive islet-antigens is associated with HLA-DQ8 transdimer-mediated autoimmune diabetes. Sci. Adv..

[28] Gravallese E.M., Firestein G.S. (2023). Rheumatoid Arthritis—Common Origins, Divergent Mechanisms. N. Engl. J. Med..

[29] Alivernini S., Firestein G.S., McInnes I.B. (2022). The pathogenesis of rheumatoid arthritis. Immunity.

[30] Taneja V., Mehra N.K., Anand C., Malaviya A.N. (1993). HLA-linked susceptibility to rheumatoid arthritis. A study of forty-one multicase families from northern India. Arthritis Rheum..

[31] Khan M.A., Kushner I., Braun W.E., Dejelo C.L., Ballou S.P. (1981). Clinical and HLA studies in multiple case families with rheumatoid arthritis. Tissue Antigens.

[32] (2007). Genome-wide association study of 14,000 cases of seven common diseases and 3,000 shared controls. Nature.

[33] Taneja V., David C.S. (2010). Role of HLA class II genes in susceptibility/resistance to inflammatory arthritis: Studies with humanized mice. Immunol. Rev..

[34] Stastny P. (1976). Mixed lymphocyte cultures in rheumatoid arthritis. J. Clin. Investig..

[35] Huizinga T.W., Amos C.I., van der Helm-van Mil A.H., Chen W., van Gaalen F.A., Jawaheer D., Schreuder G.M., Wener M., Breedveld F.C., Ahmad N. (2005). Refining the complex rheumatoid arthritis phenotype based on specificity of the HLA-DRB1 shared epitope for antibodies to citrullinated proteins. Arthritis Rheum..

[36] Taneja V., Mehra N.K., Chandershekaran A.N., Ahuja R.K., Singh Y.N., Malaviya A.N. (1992). HLA-DR4-DQw8, but not DR4-DQw7 haplotypes occur in Indian patients with rheumatoid arthritis. Rheumatol. Int..

[37] Singal D.P., Reid B., Kassam Y.B., Dsouza M., Bensen W.G., Adachi J.D. (1987). Hla-Dq Beta-Chain Polymorphism in Hla-Dr4 Haplotypes Associated with Rheumatoid-Arthritis. Lancet.

[38] Taneja V., Behrens M., Basal E., Sparks J., Griffiths M.M., Luthra H., David C.S. (2008). Delineating the role of the HLA-DR4 “shared epitope” in susceptibility versus resistance to develop arthritis. J. Immunol..

[39] Taneja V., Behrens M., Mangalam A., Griffiths M.M., Luthra H.S., David C.S. (2007). New humanized HLA-DR4-transgenic mice that mimic the sex bias of rheumatoid arthritis. Arthritis Rheum..

[40] Behrens M., Trejo T., Luthra H., Griffiths M., David C.S., Taneja V. (2010). Mechanism by which HLA-DR4 regulates sex-bias of arthritis in humanized mice. J. Autoimmun..

[41] Karlson E.W., Chibnik L.B., Cui J., Plenge R.M., Glass R.J., Maher N.E., Parker A., Roubenoff R., Izmailova E., Coblyn J.S. (2008). Associations between human leukocyte antigen, PTPN22, CTLA4 genotypes and rheumatoid arthritis phenotypes of autoantibody status, age at diagnosis and erosions in a large cohort study. Ann. Rheum. Dis..

[42] Plenge R.M., Cotsapas C., Davies L., Price A.L., de Bakker P.I., Maller J., Pe’er I., Burtt N.P., Blumenstiel B., DeFelice M. (2007). Two independent alleles at 6q23 associated with risk of rheumatoid arthritis. Nat. Genet..

[43] Jawaheer D., Li W.T., Graham R.R., Chen W., Damle A., Xiao X.L., Monteiro J., Khalili H., Lee A., Lundsten R. (2002). Dissecting the genetic complexity of the association between human leukocyte antigens and rheumatoid arthritis. Am. J. Hum. Genet..

[44] Amariuta T., Luo Y., Knevel R., Okada Y., Raychaudhuri S. (2020). Advances in genetics toward identifying pathogenic cell states of rheumatoid arthritis. Immunol. Rev..

[45] Zhang F., Wei K., Slowikowski K., Fonseka C.Y., Rao D.A., Kelly S., Goodman S.M., Tabechian D., Hughes L.B., Salomon-Escoto K. (2019). Defining inflammatory cell states in rheumatoid arthritis joint synovial tissues by integrating single-cell transcriptomics and mass cytometry. Nat. Immunol..

[46] Maeshima K., Stanford S.M., Hammaker D., Sacchetti C., Zeng L.F., Ai R., Zhang V., Boyle D.L., Aleman Muench G.R., Feng G.S. (2016). Abnormal PTPN11 enhancer methylation promotes rheumatoid arthritis fibroblast-like synoviocyte aggressiveness and joint inflammation. JCI Insight.

[47] Klareskog L., Padyukov L., Lorentzen J., Alfredsson L. (2006). Mechanisms of disease: Genetic susceptibility and environmental triggers in the development of rheumatoid arthritis. Nat. Clin. Pract. Rheumatol..

[48] Vassallo R., Luckey D., Behrens M., Madden B., Luthra H., David C., Taneja V. (2014). Cellular and humoral immunity in arthritis are profoundly influenced by the interaction between cigarette smoke effects and host HLA-DR and DQ genes. Clin. Immunol..

[49] Hill J.A., Southwood S., Sette A., Jevnikar A.M., Bell D.A., Cairns E. (2003). Cutting edge: The conversion of arginine to citrulline allows for a high-affinity peptide interaction with the rheumatoid arthritis-associated HLA-DRB1*0401 MHC class II molecule. J. Immunol..

[50] Bidkar M., Vassallo R., Luckey D., Smart M., Mouapi K., Taneja V. (2016). Cigarette Smoke Induces Immune Responses to Vimentin in both, Arthritis-Susceptible and -Resistant Humanized Mice. PLoS ONE.

[51] Wang D., Zhang J., Lau J., Wang S., Taneja V., Matteson E.L., Vassallo R. (2019). Mechanisms of lung disease development in rheumatoid arthritis. Nat. Rev. Rheumatol..

[52] Lin L., Xuan W., Luckey D., Wang S., Wang F., Lau J., Warrington K.J., Matteson E.L., Vassallo R., Taneja V. (2021). A novel humanized model of rheumatoid arthritis associated lung disease. Clin. Immunol..

[53] Catrina A.I., Deane K.D., Scher J.U. (2016). Gene, environment, microbiome and mucosal immune tolerance in rheumatoid arthritis. Rheumatology.

[54] Ebringer A., Rashid T., Wilson C. (2010). Rheumatoid arthritis, Proteus, anti-CCP antibodies and Karl Popper. Autoimmun. Rev..

[55] Meron M.K., Amital H., Shepshelovich D., Barzilai O., Ram M., Anaya J.M., Gerli R., Nicola B., Shoenfeld Y. (2010). Infectious aspects and the etiopathogenesis of rheumatoid arthritis. Clin. Rev. Allergy Immunol..

[56] de Pablo P., Dietrich T., McAlindon T.E. (2008). Association of periodontal disease and tooth loss with rheumatoid arthritis in the US population. J. Rheumatol..

[57] Hitchon C.A., El-Gabalawy H.S. (2011). Infection and rheumatoid arthritis: Still an open question. Curr. Opin. Rheumatol..

[58] Chen Y.J., Hung W.C., Chou Y.H., Lai C.H., Peng P., Jhou P.S., Tsai M.R., Sheu J.J., Yen J.H. (2022). Subgingival Microbiome in Rheumatoid Arthritis Patients with Periodontitis. Int. J. Mol. Sci..

[59] Lamba A., Taneja V. (2024). Gut microbiota as a sensor of autoimmune response and treatment for rheumatoid arthritis. Immunol. Rev..

[60] Mikuls T.R., Payne J.B., Yu F., Thiele G.M., Reynolds R.J., Cannon G.W., Markt J., McGowan D., Kerr G.S., Redman R.S. (2014). Periodontitis and Porphyromonas gingivalis in patients with rheumatoid arthritis. Arthritis Rheumatol..

[61] Biedermann L., Zeitz J., Mwinyi J., Sutter-Minder E., Rehman A., Ott S.J., Steurer-Stey C., Frei A., Frei P., Scharl M. (2013). Smoking cessation induces profound changes in the composition of the intestinal microbiota in humans. PLoS ONE.

[62] Scher J.U., Joshua V., Artacho A., Abdollahi-Roodsaz S., Ockinger J., Kullberg S., Skold M., Eklund A., Grunewald J., Clemente J.C. (2016). The lung microbiota in early rheumatoid arthritis and autoimmunity. Microbiome.

[63] Zhang X., Zhang D., Jia H., Feng Q., Wang D., Liang D., Wu X., Li J., Tang L., Li Y. (2015). The oral and gut microbiomes are perturbed in rheumatoid arthritis and partly normalized after treatment. Nat. Med..

[64] Reichert S., Haffner M., Keysser G., Schafer C., Stein J.M., Schaller H.G., Wienke A., Strauss H., Heide S., Schulz S. (2013). Detection of oral bacterial DNA in synovial fluid. J. Clin. Periodontol..

[65] Chen J., Wright K., Davis J.M., Jeraldo P., Marietta E.V., Murray J., Nelson H., Matteson E.L., Taneja V. (2016). An expansion of rare lineage intestinal microbes characterizes rheumatoid arthritis. Genome Med..

[66] Balakrishnan B., Luckey D., Wright K., Davis J.M., Chen J., Taneja V. (2023). Eggerthella lenta augments preclinical autoantibody production and metabolic shift mimicking senescence in arthritis. Sci. Adv..

[67] Wammers M., Schupp A.K., Bode J.G., Ehlting C., Wolf S., Deenen R., Kohrer K., Haussinger D., Graf D. (2018). Reprogramming of pro-inflammatory human macrophages to an anti-inflammatory phenotype by bile acids. Sci. Rep.

[68] Hamsanathan S., Gurkar A.U. (2022). Lipids as Regulators of Cellular Senescence. Front. Physiol..

[69] Flor A.C., Wolfgeher D., Wu D., Kron S.J. (2017). A signature of enhanced lipid metabolism, lipid peroxidation and aldehyde stress in therapy-induced senescence. Cell Death Discov..

[70] Montero-Melendez T., Nagano A., Chelala C., Filer A., Buckley C.D., Perretti M. (2020). Therapeutic senescence via GPCR activation in synovial fibroblasts facilitates resolution of arthritis. Nat. Commun..

[71] Han K., Singh K., Meadows A.M., Sharma R., Hassanzadeh S., Wu J., Goss-Holmes H., Huffstutler R.D., Teague H.L., Mehta N.N. (2023). Boosting NAD preferentially blunts Th17 inflammation via arginine biosynthesis and redox control in healthy and psoriasis subjects. Cell Rep. Med..

[72] Sun L., Fu J., Zhou Y. (2017). Metabolism Controls the Balance of Th17/T-Regulatory Cells. Front. Immunol..

[73] Stein L.R., Imai S. (2012). The dynamic regulation of NAD metabolism in mitochondria. Trends Endocrinol. Metab..

[74] Tang B.L. (2016). Sirt1 and the Mitochondria. Mol. Cells.

[75] Seymour B.J., Trent B., Allen B.E., Berlinberg A.J., Tangchittsumran J., Jubair W.K., Chriswell M.E., Liu S., Ornelas A., Stahly A. (2023). Microbiota-dependent indole production stimulates the development of collagen-induced arthritis in mice. J. Clin. Investig..

[76] Scher J.U., Sczesnak A., Longman R.S., Segata N., Ubeda C., Bielski C., Rostron T., Cerundolo V., Pamer E.G., Abramson S.B. (2013). Expansion of intestinal *Prevotella copri* correlates with enhanced susceptibility to arthritis. Elife.

[77] Chriswell M.E., Lefferts A.R., Clay M.R., Hsu A.R., Seifert J., Feser M.L., Rims C., Bloom M.S., Bemis E.A., Liu S.C. (2022). Clonal IgA and IgG autoantibodies from individuals at risk for rheumatoid arthritis identify an arthritogenic strain of Subdoligranulum. Sci. Transl. Med..

[78] Pianta A., Arvikar S., Strle K., Drouin E.E., Wang Q., Costello C.E., Steere A.C. (2017). Evidence of the Immune Relevance of *Prevotella copri*, a Gut Microbe, in Patients with Rheumatoid Arthritis. Arthritis Rheumatol..

[79] De Filippis F., Pasolli E., Tett A., Tarallo S., Naccarati A., De Angelis M., Neviani E., Cocolin L., Gobbetti M., Segata N. (2019). Distinct Genetic and Functional Traits of Human Intestinal *Prevotella copri* Strains Are Associated with Different Habitual Diets. Cell Host Microbe.

[80] Gupta V.K., Cunningham K.Y., Hur B., Bakshi U., Huang H., Warrington K.J., Taneja V., Myasoedova E., Davis J.M., Sung J. (2021). Gut microbial determinants of clinically important improvement in patients with rheumatoid arthritis. Genome Med..

[81] Artacho A., Isaac S., Nayak R., Flor-Duro A., Alexander M., Koo I., Manasson J., Smith P.B., Rosenthal P., Homsi Y. (2021). The Pretreatment Gut Microbiome Is Associated With Lack of Response to Methotrexate in New-Onset Rheumatoid Arthritis. Arthritis Rheumatol..

[82] Balakrishnan B., Taneja V. (2018). Microbial modulation of the gut microbiome for treating autoimmune diseases. Expert Rev. Gastroenterol. Hepatol..

[83] Rossi O., van Berkel L.A., Chain F., Tanweer Khan M., Taverne N., Sokol H., Duncan S.H., Flint H.J., Harmsen H.J.M., Langella P. (2016). Faecalibacterium prausnitzii A2-165 has a high capacity to induce IL-10 in human and murine dendritic cells and modulates T cell responses. Sci. Rep..

[84] Yan F., Polk D.B. (2010). Disruption of NF-kappaB signalling by ancient microbial molecules: Novel therapies of the future?. Gut.

[85] Shi Z., Li M., Zhang C., Li H., Zhang Y., Zhang L., Li X., Li L., Wang X., Fu X. (2024). Butyrate-producing *Faecalibacterium prausnitzii* suppresses natural killer/T-cell lymphoma by dampening the JAK-STAT pathway. Gut.

[86] He J., Chu Y., Li J., Meng Q., Liu Y., Jin J., Wang Y., Wang J., Huang B., Shi L. (2022). Intestinal butyrate-metabolizing species contribute to autoantibody production and bone erosion in rheumatoid arthritis. Sci. Adv..

[87] Coccia C., Bonomi F., Lo Cricchio A., Russo E., Peretti S., Bandini G., Lepri G., Bartoli F., Moggi-Pignone A., Guiducci S. (2024). The Potential Role of Butyrate in the Pathogenesis and Treatment of Autoimmune Rheumatic Diseases. Biomedicines.

[88] Pineda M.D., Thompson S.F., Summers K., de Leon F., Pope J., Reid G. (2011). A randomized, double-blinded, placebo-controlled pilot study of probiotics in active rheumatoid arthritis. Med. Sci. Monit..

[89] Zamani B., Golkar H.R., Farshbaf S., Emadi-Baygi M., Tajabadi-Ebrahimi M., Jafari P., Akhavan R., Taghizadeh M., Memarzadeh M.R., Asemi Z. (2016). Clinical and metabolic response to probiotic supplementation in patients with rheumatoid arthritis: A randomized, double-blind, placebo-controlled trial. Int. J. Rheum. Dis..

[90] Kato I., Endo-Tanaka K., Yokokura T. (1998). Suppressive effects of the oral administration of *Lactobacillus casei* on type II collagen-induced arthritis in DBA/1 mice. Life Sci..

[91] Bungau S.G., Behl T., Singh A., Sehgal A., Singh S., Chigurupati S., Vijayabalan S., Das S., Palanimuthu V.R. (2021). Targeting Probiotics in Rheumatoid Arthritis. Nutrients.

[92] Balakrishnan B., Luckey D., Bodhke R., Chen J., Marietta E., Jeraldo P., Murray J., Taneja V. (2021). *Prevotella histicola* Protects from Arthritis by Expansion of *Allobaculum* and Augmenting Butyrate Production in Humanized Mice. Front. Immunol..

[93] Balakrishnan B., Luckey D., Bodkhe R., Taneja V. (2020). *Prevotella histicola* treatment reduces arthritic pain and partially normalizes gut microbiota and metabolites. J. Immunol..

[94] Balakrishnan B., Johnson S., Luckey D., Marietta E., Murray J., Taneja V. (2024). Small intestinal derived *Prevotella histicola* simulates biologic as a therapeutic agent. Sci. Rep..

[95] Arumugam M., Raes J., Pelletier E., Le Paslier D., Yamada T., Mende D.R., Fernandes G.R., Tap J., Bruls T., Batto J.M. (2011). Enterotypes of the human gut microbiome. Nature.

[96] Zhao L., Zhang F., Ding X., Wu G., Lam Y.Y., Wang X., Fu H., Xue X., Lu C., Ma J. (2018). Gut bacteria selectively promoted by dietary fibers alleviate type 2 diabetes. Science.

[97] Hu Y., Costenbader K.H., Gao X., Al-Daabil M., Sparks J.A., Solomon D.H., Hu F.B., Karlson E.W., Lu B. (2014). Sugar-sweetened soda consumption and risk of developing rheumatoid arthritis in women. Am. J. Clin. Nutr..

[98] Hu Y., Sparks J.A., Malspeis S., Costenbader K.H., Hu F.B., Karlson E.W., Lu B. (2017). Long-term dietary quality and risk of developing rheumatoid arthritis in women. Ann. Rheum. Dis..

[99] Khanna S., Jaiswal K.S., Gupta B. (2017). Managing Rheumatoid Arthritis with Dietary Interventions. Front. Nutr..

[100] Chasov V., Gilyazova E., Ganeeva I., Zmievskaya E., Davletshin D., Valiullina A., Bulatov E. (2024). Gut Microbiota Modulation: A Novel Strategy for Rheumatoid Arthritis Therapy. Biomolecules.

[101] Dong Y., Yao J.L., Deng Q.Y., Li X.X., He Y.Y., Ren X.Y., Zheng Y., Song R.L., Zhong X.J., Ma J.M. (2023). Relationship between gut microbiota and rheumatoid arthritis: A bibliometric analysis. Front. Immunol..

